# Public Education

**Published:** 1921-02-26

**Authors:** 


### The Hospital

The Workers' Newspaper of Administrative Medicine and Institutional Life. Administration. National Insurance and Health.

No. 1812, Vol. LXIX. SATURDAY, FEBRUARY 26, 1921. PRICE TWOPENCE

### PUBLIC EDUCATION.

A short time ago we emphasised the fact that it must at all costs be brought home to all classes that they have a definite duty to perform in subscribing to the support of the voluntary hospitals. Of course, there is a great difference between the provision of expert hospital treatment and the gradual establishment of a better average hygienic standard of living. We see it stated in an American contemporary that, "with certain limitations a people can have such hygienic conditions as they choose to buy," and from this point of view also the people must realise that they have their definite share to pay. In the next few months they will again read proposals, criticisms, and counter-criticisms of a variety of measures which the Health Ministry has under consideration, and it is sincerely to be hoped that there is sufficient public enlightenment on the importance of these matters to prevent their indiscriminate mutilation by an anti-waste campaign. There is, however, considerable danger that the recent boom in health matters may have tended to confuse those masses who are, above all others, in need of greatly improved average health. At one moment they were told by the press which they habitually read that the one crying national health need is greater financial help for the great voluntary hospitals. At the next, they heard repeatedly of the magnificent future which lies before preventive medicine. Ideals in every direction, vaguely mingled with accounts of much-needed research endowment, have been paraded before them as the essentials and the foundation of a great new era in which the health returns of Great Britain will steadily and marvellously improve.

Nevertheless, the situation, as so aptly described by Sir George Newman in his address on "The Place of Public Opinion in Preventive Medicine," has still to be cleared up—cleared up not from the point of view of the intellectual, or even that of the averagely educated man, but from the natural standpoint of the man who is not habitually a brain-worker. Modern state-controlled education has made even the most extreme class of manual worker far more easy of approach than he was twenty years ago. But the point upon which we have our doubts is whether the lay press, the great, and, in relative power, the best, medium through which a really efficient beginning in self-organised hygiene can be made, is not by the very profusion of its so-called illustrations often defeating the ends it would serve.

International control of disease, inter-county control, and interest in the health of all the people from the general welfare point of view are functions which the actual public health services, aided by legislation, are well able to perform; but, as so many health officers complain, without real understanding on the part of the people, official care is inevitably met by that attitude which some time ago prompted the remark that the people would be "bossed by doctors from the cradle to the grave." In these pages we recently endeavoured to summarise as the "Will to Health" the necessary state of mind, and that which will meet administrative endeavours half-way. In certain special types of disease we admit that sufficient enlightenment—or, shall we say, concern?—has been produced not only materially to aid organised curative measures, but also to some extent to develop reasonable prevention. But these have been in outstanding instances which lent themselves readily to the methods of popular propaganda. Thousands of minor health fallacies and thousands of insidious influences conducing to disease remain impossible of reasonable explanation in average daily paper comment, and before they can be in part even eliminated there must be a really generalised sympathy between the health services and the people. If this sympathy be once developed the members of the health service can themselves be relied upon to do in one year what a lay paper may fail to do in a generation. We know of more than one health officer who by courtesy of the local press has carried out a campaign under his own authorship and guidance. The results speak for themselves.

Under the new Ministry there is every centralised advantage for the organisation of such propaganda. But cannot the facts be brought yet more and more insistently to the working public—and in such form that real instruction is provided as well as refutation of the absurd criticisms so prevalent lately? Loud press comment against a relatively silent health service can have but one effect upon the restless public of to-day.

It is one thing to urge the financial support of organised medicine by attempting the extremely difficult task of demonstrating to the masses the meaning of its highest and most technical ideas, but quite another—and, indeed, a better, we believe—deliberately to educate and interest the working man in the real meaning of the activity and care which the health authorities exert.

